# Chronic inflammatory pain alters alcohol-regulated frontocortical signaling and associations between alcohol drinking and thermal sensitivity

**DOI:** 10.1016/j.ynpai.2020.100052

**Published:** 2020-09-15

**Authors:** M. Adrienne McGinn, Kimberly N. Edwards, Scott Edwards

**Affiliations:** a Neurobiology of Addiction Section, National Institute on Drug Abuse IRP, United States; b Department of Physiology, LSU Health-New Orleans, United States; c Alcohol & Drug Abuse Center of Excellence, LSU Health-New Orleans, United States; d Neuroscience Center of Excellence, LSU Health-New Orleans, United States; e Comprehensive Alcohol-HIV/AIDS Research Center, LSU Health-New Orleans, United States

**Keywords:** Alcohol, Central amygdala, ERK, Glucocorticoid receptor, Pain, Prefrontal cortex

## Abstract

Alcohol use disorder (AUD) is a chronic, relapsing psychiatric disorder that is characterized by the emergence of negative affective states. The transition from recreational, limited intake to uncontrolled, escalated intake is proposed to involve a transition from positive to negative reinforcement mechanisms for seeking alcohol. Past work has identified the emergence of significant hyperalgesia/allodynia in alcohol-dependent animals, which may serve as a key negative reinforcement mechanism. Chronic pain has been associated with enhanced extracellular signal-regulated kinase (ERK) activity in cortical and subcortical nociceptive areas. Additionally, both pain and AUD have been associated with increased activity of the glucocorticoid receptor (GR), a key mediator of stress responsiveness. The objectives of the current study were to first determine relationships between thermal nociceptive sensitivity and alcohol drinking in male Wistar rats. While inflammatory pain induced by complete Freund’s adjuvant (CFA) administration did not modify escalation of home cage drinking in animals over four weeks, the relationship between drinking levels and hyperalgesia symptoms reversed between acute (1 week) and chronic (3–4 week) periods post-CFA administration, suggesting that either the motivational or analgesic effects of alcohol may be altered over the time course of chronic pain. We next examined ERK and GR phosphorylation in pain-related brain areas (including the central amygdala and prefrontal cortex subregions) in animals experiencing acute withdrawal from binge alcohol administration (2 g/kg, 6 h withdrawal) and CFA administration (four weeks) to model the neurobiological consequences of binge alcohol exposure in the context of pain. We observed a significant interaction between alcohol and pain state, whereby alcohol withdrawal increased ERK phosphorylation across all four frontocortical areas examined, although this effect was absent in animals experiencing chronic inflammatory pain. Alcohol withdrawal also increased GR phosphorylation across all four frontocortical areas, but these changes were not altered by CFA. Interestingly, we observed significant inter-brain regional correlations in GR phosphorylation between the insula and other regions investigated only in animals exposed to both alcohol and CFA, suggesting coordinated activity in insula circuitry and glucocorticoid signaling in this context. The results of these studies provide a greater understanding of the neurobiology of AUD and will contribute to the development of effective treatment strategies for comorbid AUD and pain.

## Highlights

• Associations between alcohol drinking and pain sensitivity change over time.
• Alcohol withdrawal-induced changes in ERK phosphorylation depend on pain status.
• Exposure to both alcohol and pain synchronize brain glucocorticoid signaling.

## 1 Introduction

Chronic pain impacts over 20% of the global population (Dahlhamer et al., 2018) and exacts a heavy toll on both physical and mental health (Elman et al., 2013). Inflammatory pain conditions comprise the majority of chronic pain complaints, with arthritis representing the leading cause of disability among adults (Hootman et al., 2016). Chronic pain patients also report higher levels of alcohol drinking and suffer from alcohol use disorder (AUD, formerly alcohol dependence) at rates higher than the general population (Hoffmann et al., 1995, Vowles et al., 2018). While it has long been observed that alcohol serves as an effective analgesic in the face of pain (Wolff et al., 1942), heavy alcohol use itself often exacerbates nociceptive hypersensitivity in both humans and animal models as part of a broader alcohol withdrawal syndrome (Gatch and Lal, 1999, Jochum et al., 2010, Edwards et al., 2012, Fu et al., 2015). Such data suggest that frequent drinking in individuals suffering from AUD may be motivated in part by a desire to alleviate such enhanced nociceptive sensitivity, or hyperalgesia (Zale et al, 2015). At the preclinical level, partial evidence for this relationship comes from a recent study where hyperalgesia symptoms in alcohol-dependent rats were attenuated following either alcohol self-administration or experimenter-administered alcohol (Roltsch Hellard et al., 2017). In humans, self-reports of alcohol use specifically for pain management are common (e.g., Riley and King, 2009), while thermal pain-related increases in alcohol craving have been specifically shown to be mediated by pain-related negative affect (Moskal et al., 2018). Moreover, problem drinkers at risk for AUD not only report more severe pain symptoms compared to non-drinkers, but also report a higher incidence of using alcohol to manage their pain (Brennan et al., 2005). Interestingly, the analgesic effects of alcohol in a cold pressor test (CPT) were stronger in alcoholics vs. non-alcoholics (Cutter et al., 1976), while CPT-induced alcohol craving predicts future alcohol use in alcohol-dependent individuals (Brady et al., 2006). These findings indicate that self-medication and negative reinforcement mechanisms related to pain-related drinking could underlie the persistence or exacerbation of AUD over time (Apkarian et al., 2013, McGinn and Edwards, 2016, McDermott et al., 2018).

Strong evidence demonstrates that the neurobiological substrates associated with alcohol reward overlap considerably with the supra-spinal (neural) substrates of the emotional aspects of pain processing (Koob and Volkow, 2010, Egli et al., 2012, Borsook et al., 2016, Robins et al., 2019). Specifically, the affective component of pain appears to be strongly mediated by the central amygdala (CeA; Neugebauer, 2015) along with select frontocortical brain centers including the medial prefrontal cortex, cingulate cortex, and insular cortex (Vogt, 2005a, Vogt, 2005b, George and Koob, 2010, Zhuo, 2016). Chronic pain is associated with specific neuronal plasticity throughout this network, including functional increases in activation of extracellular signal-regulated kinase (ERK) signaling that closely associates with the affective expression of pain (Johansen et al., 2001, Wei and Zhuo, 2008, Cao et al., 2009). Neuroadaptations in ERK phosphorylation status are also evident in animal models of alcohol withdrawal and AUD (Sanna et al., 2002, Zamora-Martinez and Edwards, 2014), indicating that ERK may serve as a useful biomarker of neuronal activity to interrogate how pain and alcohol withdrawal interact to dysregulate brain circuitry.

The neurobiological intersection of pain and alcohol reinforcement may also represent valuable opportunities for novel, receptor-mediated therapeutic avenues targeting each condition (Edwards et al., 2020). Importantly, dysregulated stress signaling is common to both conditions (LeBlanc et al., 2015). For example, stress-related glucocorticoid receptor (GR) activity is implicated in the development and maintenance of chronic pain states (Blackburn-Munro and Blackburn-Munro, 2003, Madalena and Lerch, 2017) as both stress and GR activation exacerbate pain-related behaviors. Consistent with these findings, GR antagonism or adrenalectomy can reduce and even prevent the development of hyperalgesia-like symptoms (Wang et al., 2005, Dina et al., 2008, Alexander et al., 2009, Le Coz et al., 2014). Importantly, functional increases in central nervous system GR phosphorylation and activity promote the development of escalated drinking in animal models of AUD, while GR antagonism with mifepristone reduces symptoms of both craving and subsequent heavy drinking in humans suffering from AUD (Vendruscolo et al., 2015).

To better understand the biobehavioral relationships between alcohol use and chronic pain, the objectives of the current study were to first longitudinally examine relationships between recent alcohol drinking and thermal nociceptive sensitivity in male Wistar rats over a four-week course of inflammatory pain using the complete Freund’s adjuvant (CFA) model. We then investigated neuroadaptations produced by acute withdrawal from a binge-like exposure to alcohol in the context of a chronic inflammatory pain state. We focused on alterations in ERK and GR phosphorylation levels within subcortical (central amygdala) and cortical (anterior cingulate, insula, medial prefrontal cortex) brain areas representing potential points of intersection between central brain nociceptive processing and alcohol reinforcement (Egli et al., 2012, Edwards et al., 2020).

## 2 Material and methods

### 2.1 Animals

Adult male Wistar rats were purchased from Charles River and weighed 200–300 g at the time of arrival. Rats were pair-housed and given *ad libitum* access to food (Purina Rat Chow, Ralston Purina, St. Louis, MO) and water throughout behavioral training and testing. Rats were maintained on a reverse 12-hour light/dark cycle (lights off at 8:00 am). Rats were handled regularly and given one week to acclimate to the colony room prior to the start of experimental procedures. All animal care, use, and procedures in this study were approved by the Institutional Animal Care and Use Committee of Louisiana State University Health Sciences Center (LSUHSC) and were in accordance with the National Institute of Health guidelines.

### 2.2 Complete Freund’s Adjuvant-Induced chronic inflammatory pain

To produce a sustained, localized inflammatory state, 150 μL of 50% complete Freund’s adjuvant (Sigma) in saline was injected subcutaneously into the intraplantar surface of the left hind paw using a 26-gauge needle. This produced visible inflammation that resulted in mechanical and thermal hypersensitivity as measured by the von Frey and cold plate methods (respectively) described below. Inflammation and mechanical hypersensitivity is reported to last up to four weeks (Nagakura et al., 2003) but has been observed for up to 8 weeks in the Edwards Lab. Control rats were injected with 150 μL of 0.9% saline into the left hind paw that produced no inflammation or pain hypersensitivity.

### 2.3 Intermittent Access, 2-Bottle choice 20% alcohol drinking

An intermittent-access alcohol drinking procedure was utilized that facilitates an escalation of alcohol drinking over time, as described in Simms et al. (2008). Rats were single-housed with ad libitum access to food and water and two bottles in each cage at all times. On Mondays, Wednesdays, and Fridays one bottle containing 20% w/v ethanol was placed in the home cage 15 min after the start of the rats’ dark cycle (8:15 AM). After 30 min the alcohol and H_2_O bottles were weighed and placed back into the cage. The alcohol and H_2_O bottles were weighed again 24 h later, and the alcohol bottle was replaced with a H_2_O bottle (Tuesdays, Thursdays, and Saturdays). On alcohol-drinking days, the position of the alcohol bottle was alternated to prevent the development of a side-preference. Rat bodyweights were taken every day and alcohol consumption was calculated as grams of alcohol consumed per kilogram of body weight. The first drinking session began 3 days post-CFA treatment.

### 2.4 Cold plate thermal sensitivity testing

Animals were tested for cold allodynia using the Ugo Basile Hot/Cold Plate (Stoelting Co., Wood Dale, IL). The test surface was set to 3 °C, which is a non-noxious temperature. Animals were placed onto the test surface that was enclosed by a plexiglass cylinder. The nociceptive response was defined as the paw withdrawal latency in seconds. A sharp withdrawal of the left hindpaw was considered a positive response. The maximal test time was set at 120 s. Uninjured animals never displayed a nociceptive response within the 120-second test period.

### 2.5 Von Frey filament mechanical sensitivity testing

Mechanical sensitivity was determined by obtaining hind paw withdrawal thresholds, similar to what is described in Edwards et al. (2012). Animals were first acclimated for 10 min to individual plexiglass compartments set on top of a mesh stand. A series of nylon von Frey filaments (North Coast Medical) were applied perpendicularly to the plantar surface of the hind paw until they buckled for 2 s, and a sharp withdrawal of the stimulated hind paw before or within the 2 s indicated a positive response. Testing was initiated with the filament corresponding to 15 g of force and continued in accordance with the up-and-down method (Dixon, 1980). The 50% paw withdrawal threshold was determined by the formula Xf + kδ, where Xf = last von Frey filament used, k = Dixon value corresponding to response pattern, and δ = mean difference between stimuli. Baseline paw withdrawal thresholds were obtained prior to CFA administration and/or alcohol exposure for all experimental groups.

### 2.6 Binge alcohol exposure & withdrawal

Animals were injected interperitoneally (I.P.) with either saline (1 ml/kg) or alcohol (2 g/kg of 15% w/v ethanol solution) and euthanized 6 h later, a time point at which blood alcohol levels are negligible (Morse et al., 2000). This procedure was employed as a model of “acute alcohol dependence”, where withdrawal from a single 2 g/kg dose produces affective symptoms comparable to what is observed in rodent models of chronic alcohol dependence (Schulteis and Liu, 2006). Rats underwent binge alcohol exposure and withdrawal 4 weeks after CFA or saline administration. One day prior to sacrifice, all rats received a 1 ml/kg I.P. injection of saline to habituate them to the injection procedure.

### 2.7 Western blot analysis

Animals used for western blot analysis were sacrificed by decapitation under light isofluorane anesthesia. Brains were rapidly dissected, snap-frozen in −30 °C isopentane, and stored at −80 °C until microdissection. Regional tissue samples were taken from frozen coronal brain sections (0.5 mm thick) using 13–16 gauge punches on a cryostat. Tissue was collected from anatomical regions in accordance with Paxinos and Watson (1998). Tissue punches were stored at −80 °C until homogenization. Tissue samples were homogenized by adding lysis buffer (320 mm sucrose, 5 mm HEPES, 1 mm EGTA, 1 mm EDTA, and 1% SDS, with protease inhibitor cocktail and phosphatase inhibitor cocktails II and III diluted 1:100; Sigma-Aldrich) followed by brief sonication, then heated at 100 °C for 5 min. Protein concentration was determined by a colorimetric Lowry assay (DC™ protein assay, Bio-Rad). Samples were stored in 20 µg/µl protein aliquots at −80 °C until use for Western analysis as previously described (McGinn et al., 2016). Briefly, samples underwent SDS–polyacrylamide gel electrophoresis on 8% acrylamide gels using a Tris/Glycine/SDS buffer system (Bio-Rad), followed by overnight electrophoretic transfer to PVDF membranes (ThermoFisher). After the transfer, membranes were blocked in 5% non-fat milk for 1 h at room temperature, then incubated in primary antibody in 2.5% milk overnight at 4 °C. Primary antibodies included phospho-ERK (1:5000 Cat #9106), total ERK (1:20,000 Cat #9102), phospho-GR^Ser211^ (1:1000, Cat #4161, Cell Signaling), total GR (1:1000, Cat #MA1-510, Invitrogen), and GAPDH (1:1 million, Cat #AB9485, Abcam). Membranes were washed in TTBS three times for 10 min at room temperature, labeled with species-specific peroxidase-conjugated secondary antibody (1:10,000 in TTBS; Bio-Rad) for 1 h at room temperature, again washed 3x, then finally detected by chemiluminescence (SuperSignal West Pico; ThermoFisher) with x-ray film (Denville Scientific). Immunoreactivity was quantified by densitometry using ImageJ (NIH) Protein densitometry values were normalized to the loading control protein GAPDH densitometry values. For phosphoproteins, the phosphoprotein blots were stripped for 40 min at room temperature (Restore, ThermoFisher) and were re-probed for total protein levels. Phosphoprotein densitometry values were normalized to total protein densitometry values to generate phosphorylated:total protein ratio values for statistical comparison. These values were further normalized to loading control GAPDH if significant changes were observed in total protein between experimental groups. Densitized values were expressed as a percentage of the mean of control values for each gel to normalize data across blots.

### 2.8 Statistical analysis

All data are presented as mean ± SEM. Statistical analysis was performed in Prism 8 (GraphPad Software). Data were analyzed two-way ANOVAs with Tukey’s multiple comparisons tests as *post hoc* analyses, or one- or two-way repeated measures ANOVAs with Sidak’s multiple comparisons tests as *post hoc* analyses as indicated in the text except where it is indicated otherwise. Statistical significance was set as p < 0.05.

## 3 Results

### 3.1 CFA-treated animals exhibit prolonged thermal hyperalgesia, but consume similar total amounts of alcohol compared to control (saline-treated) animals and escalate alcohol drinking over time at a similar rate

**Fig. 1:**
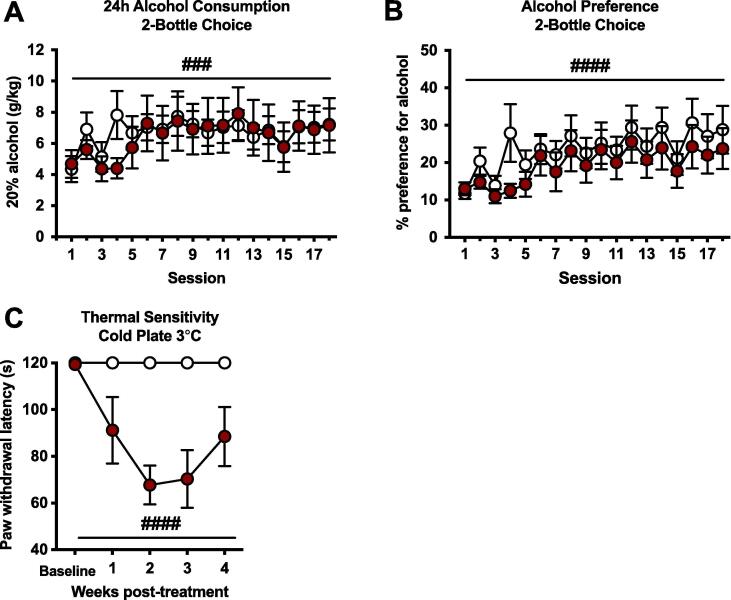
Chronic inflammatory pain, thermal hyperalgesia, and alcohol consumption and preference over time in a 2-bottle choice intermittent access drinking procedure. There were no significant differences between treatment groups in (A) alcohol consumption over 24 h, or (B) preference for alcohol over water. There was a significant escalation of both alcohol consumption (###p < 0.001 main effect of time) and preference (####p < 0.0001 main effect of time) over the four-week period. (C) CFA-treated male rats displayed cold hyperalgesia over four weeks post-CFA injection (####p < 0.0001 main effect of group). Data are represented as mean ± SEM. Control, white (n = 7); CFA-treated, red (n = 7). (For interpretation of the references to colour in this figure legend, the reader is referred to the web version of this article.)

Animals undergoing a home cage, intermittent access, two-bottle choice alcohol drinking procedure displayed a significant escalation of alcohol drinking over 18 drinking sessions (Fig. 1A). A two-way repeated-measures ANOVA of mean 24 h alcohol consumption indicated a significant effect of session (F_17,204_ = 2.920, p = 0.0002). However, there was no effect of CFA treatment (F_1,12_ = 0.033, p = 0.860) or a group × session interaction (F_17,204_ = 0.882, p = 0.596) on the total amount of alcohol consumed over the entire 24 h period of access to alcohol. Similarly, there was a main effect of session in terms of alcohol preference over water (F_17,204_ = 5.761, p < 0.0001; Fig. 1B), indicating a similar increase in preference for alcohol over time in both groups. CFA-treated animals exhibited significant thermal hyperalgesia (Fig. 1C) as measured by the cold plate test over the course of the four weeks of testing (main effect of group: F_1,12_ = 38.52, p < 0.0001; significant group × time interaction: F_4,48_ = 3.802, p = 0.009).

### 3.2 The relationship between alcohol consumption and thermal sensitivity changes over time in CFA-treated animals

**Fig. 2:**
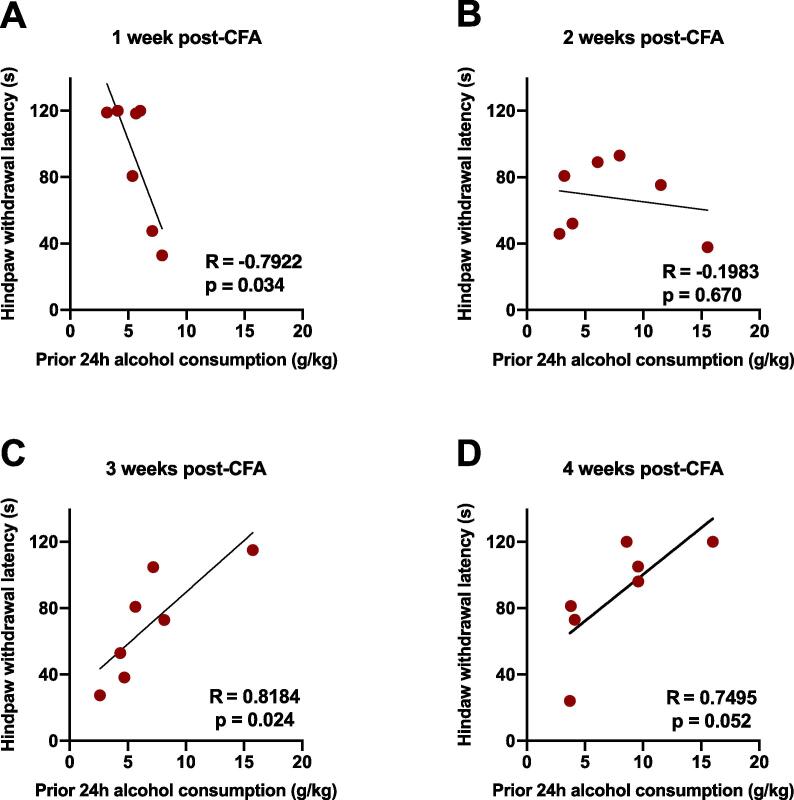
Relationship between thermal hyperalgesia and alcohol consumption in animals with chronic inflammatory pain over four weeks. (A) Hindpaw-withdrawal latency negatively correlated with prior 24 h alcohol consumption 1 week post-CFA treatment (R = −0.7922, p = 0.034); (B) There was no correlation between hindpaw-withdrawal latency and prior 24-hour alcohol consumption 2 weeks post-CFA treatment (R = −0.1983, p = 0.670); (C) Hindpaw-withdrawal latency positively correlated with prior 24-hour alcohol consumption 3 weeks post-CFA treatment (R = 0.8184, p = 0.024); (D) Hindpaw-withdrawal latency positively correlated with prior 24-hour alcohol consumption 4 weeks post-CFA treatment (R = 0.7495, p = 0.052). Data were analyzed using linear regression. CFA-treated, red (n = 7). (For interpretation of the references to colour in this figure legend, the reader is referred to the web version of this article.)

Although no main effect of CFA treatment on either the escalation of alcohol drinking or alcohol preference was found, we next examined the relationship between paw withdrawal latencies on the cold plate thermal nociceptive assay and the amount of alcohol consumed over the previous 24 h access period (Fig. 2). Tests were conducted several hours following the most recent 24 h drinking session during the weeks indicated. One week after CFA administration, hindpaw withdrawal latencies of the CFA group negatively correlated with prior 24 h alcohol consumption (R^2^ = 0.628, p = 0.034, Fig. 2A), such that animals showing more severe hyperalgesia had consumed more alcohol the day prior to testing. However, two weeks following CFA administration, there was no directional relationship between paw withdrawal latencies and recent alcohol consumption (R^2^ = 0.039, p = 0.670, Fig. 2B). Interestingly, and in contrast to 1-week post-CFA findings, at both 3 and 4 weeks post-CFA administration, hindpaw withdrawal latencies of the CFA-treated group were positively correlated with recent (prior 24 h) alcohol consumption, such that animals showing less severe hyperalgesia had consumed more alcohol prior to testing (R^2^ = 0.670, p = 0.024, Fig. 2C; R^2^ = 0.562, p = 0.052, Fig. 2D).

### 3.3 Withdrawal from a single binge alcohol exposure increases ERK phosphorylation across several frontocortical brain regions, although this effect is significantly dampened in the context of chronic inflammatory pain

**Fig. 3:**
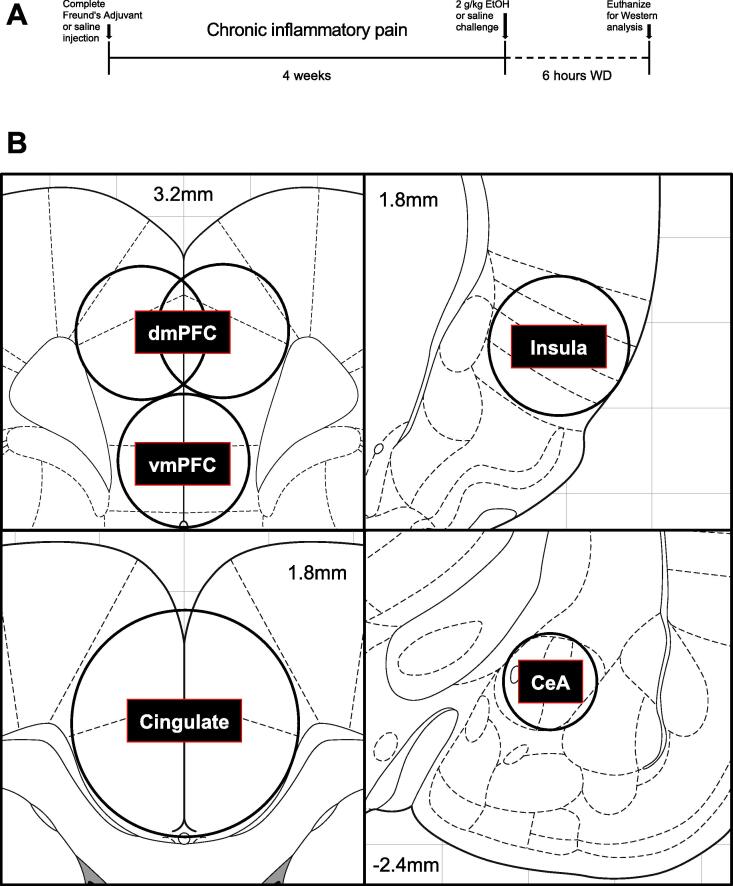
Study timeline and regional brain dissections for molecular experiments.

**Fig. 4:**
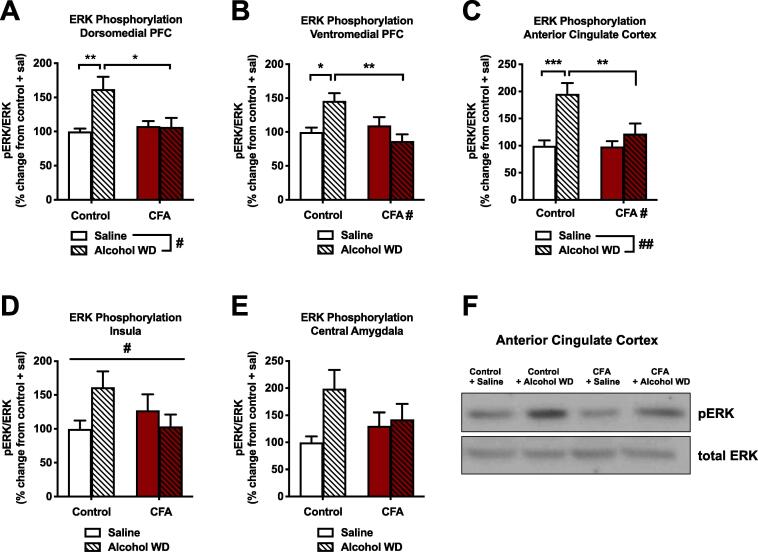
Interaction of chronic inflammatory pain and binge alcohol withdrawal on ERK phosphorylation. Binge alcohol exposure and withdrawal increased ERK phosphorylation in control but not CFA-treated rats in the (A) dorsomedial PFC, (B) ventromedial PFC, and (C) anterior cingulate cortex. (D) There was a significant interaction for CFA treatment and acute alcohol withdrawal on pERK in the insula, but no significant differences between groups. (E) pERK in the central amygdala was not significantly altered by CFA treatment or acute alcohol withdrawal. (F) Representative Western blots of pERK and total ERK in the anterior cingulate cortex. Data were analyzed using 2-way ANOVA and Tukey’s post hoc tests. Data are represented as mean ± SEM. Saline control + saline, solid white (n = 9); Saline control + acute alcohol WD, white striped (n = 11); CFA + saline, solid red (n = 9); CFA + acute alcohol WD, red striped (n = 9). (For interpretation of the references to colour in this figure legend, the reader is referred to the web version of this article.)

In a separate cohort of animals, we next examined neurobiological interactions between binge alcohol exposure and withdrawal and chronic (4-week) CFA treatment (Fig. 3A). Animals were treated with intra-plantar CFA or saline. Four weeks later, these two groups were further divided into animals receiving saline (IP, 1 ml/kg) or alcohol (IP, 2 g/kg) and sacrificed six hours later, a time point where blood alcohol levels return to near zero (Schulteis and Liu, 2006). These treatments were preceded on the two days prior with habituating IP injections of saline (1 ml/kg). Before the day of sacrifice, decreased paw withdrawal thresholds (indicative of hyperalgesia, determined via von Frey testing) were confirmed in the CFA-treated animals (11.56 ± 2.53 g) vs. saline-treated animals (44.21 ± 4.86 g). We first examined phosphorylation of ERK as a general marker of activity in the brain regions of interest (Fig. 3B). In the dorsomedial prefrontal cortex (dmPFC, Fig. 4A), we observed a main effect of alcohol withdrawal (F_1,34_ = 5.794, p = 0.0217) but also an interaction of alcohol withdrawal and CFA (F_1,34_ = 6.248, p = 0.0174), indicating that ERK phosphorylation increases in CFA-treated animals (6.8%) were significantly less than increases in saline-treated animals (62.4%) according to Tukey’s *post hoc* testing (q_34_ = 4.483). We found a similar pattern in the ventromedial prefrontal cortex (vmPFC, Fig. 4B), where an interaction of alcohol withdrawal and CFA (F_1,34_ = 11.05, p = 0.0021) indicated that ERK phosphorylation increases in CFA-treated animals (13.1%) were significantly less than increases in saline-treated animals (46.0%) according to Tukey’s *post hoc* testing (q_34_ = 5.85). In the anterior cingulate cortex (Fig. 4C), 2-way ANOVA revealed main effects of both alcohol withdrawal (F_1,34_ = 13.66, p = 0.0008) and CFA treatment (F_1,34_ = 5.393, p = 0.0263), as well as another interaction between factors (F_1,34_ = 5.035, p = 0.0315) indicating that ERK phosphorylation increases in CFA-treated animals (22%) were significantly less than increases in saline-treated animals (96%) according to Tukey’s *post hoc* testing (q_34_ = 4.679). Finally, the opposing interaction between factors was also observed in the insular cortex (Fig. 4D, F1_34_ = 4.313, p = 0.0454), with a modest trend for interaction occurring in the central amygdala (Fig. 4E, F1_34_ = 2.506, p = 0.1227). Representative blots for the anterior cingulate cortex are shown in Fig. 4F. In summary, alcohol withdrawal produced widespread increases in ERK phosphorylation that appeared to be blunted in animals with a history of inflammatory pain across all frontocortical brain regions examined.

### 3.4 Withdrawal from binge alcohol exposure increases GR phosphorylation across frontocortical brain regions, although this effect is not influenced by chronic inflammatory pain

**Fig. 5:**
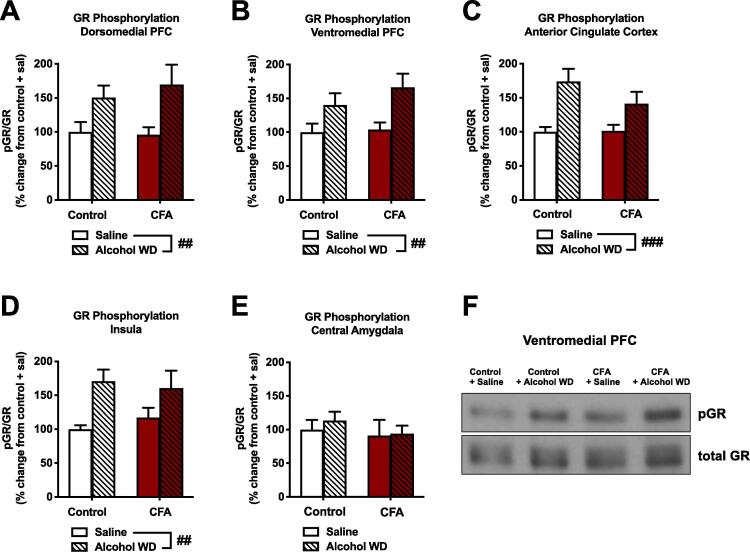
The interaction of chronic inflammatory pain and binge alcohol withdrawal on glucocorticoid receptor phosphorylation. Binge alcohol exposure and withdrawal increased GR phosphorylation (Ser232 in both control and CFA-treated animals in the (A) dorsomedial PFC, (B) ventromedial PFC, (C) anterior cingulate cortex, and (D) insula (##p < 0.01, ###p < 0.001 main effect of group). (E) In the CeA, there was no effect of CFA treatment or acute alcohol WD on GR Ser232 phosphorylation. (F) Representative Western blots of pGR and total GR in the ventromedial PFC. Data were analyzed using 2-way RM ANOVA and Sidak's multiple comparisons test (B) or 2-way ANOVA and Tukey’s post hoc tests (C-G). Data are represented as mean ± SEM. Saline control + saline, solid white (n = 9); Saline control + acute alcohol WD, white striped (n = 11); CFA + saline, solid red (n = 9); CFA + acute alcohol WD, red striped (n = 9). (For interpretation of the references to colour in this figure legend, the reader is referred to the web version of this article.)

We also examined the phosphorylation status of glucocorticoid receptors at serine 232, a marker of nuclear localization and transcriptional activation. In both the dmPFC (Fig. 5A) and vmPFC (Fig. 5B), we observed main effects of alcohol withdrawal (F_1,34_ = 10.68, p = 0.0025; F_1,34_ = 10.74, p = 0.0024, respectively), but no effect of CFA or interactions of alcohol withdrawal × CFA. Similar patterns were seen in the anterior cingulate cortex (Fig. 5C) and insular cortex (Fig. 5D), where we also observed a main effect of alcohol withdrawal to increase pGR^S232^ (F_1,34_ = 15.49, p = 0.0004; F_1,34_ = 10.85, p = 0.0023, respectively), but no alcohol withdrawal × CFA interaction. In contrast to cortical areas examined, a 2-way ANOVA of pGR^S232^ levels in the central amygdala (Fig. 5E) determined no significant main effect of alcohol withdrawal, CFA treatment, or an alcohol withdrawal × CFA interaction. Representative blots for the vmPFC are shown in Fig. 5F.

### 3.5 Positive correlations in GR phosphorylation levels between the insula and other nociceptive brain areas in animals exposed to both CFA and binge alcohol withdrawal

**Fig. 6:**
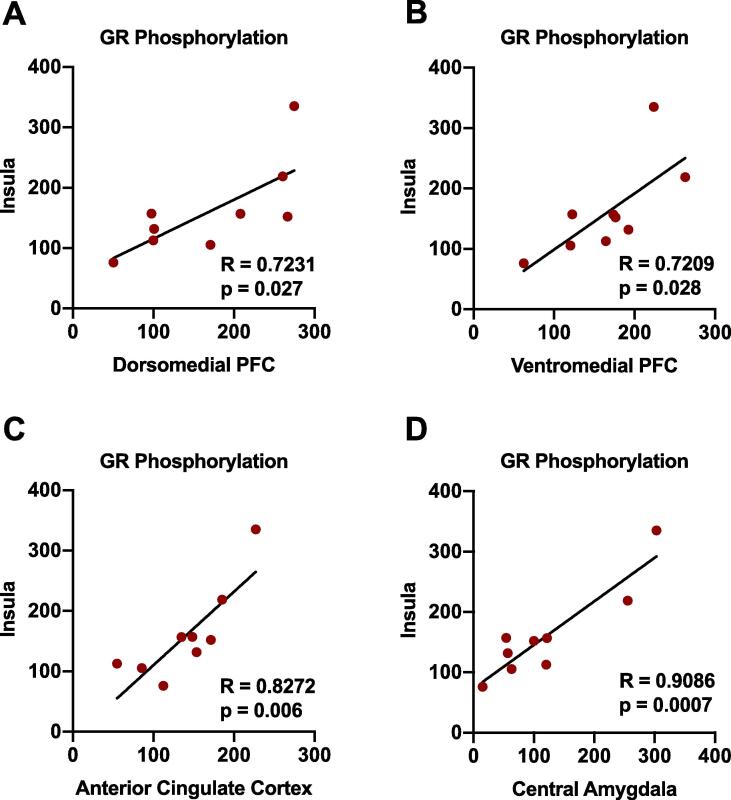
Inter-brain regional correlations between the insular cortex and other nociceptive brain regions. Significant positive correlations are evident in GR phosphorylation levels between the insula and all other areas investigated (dmPFC, vmPFC, anterior cingulate cortex, and central amygdala) in animals exposed to both binge alcohol withdrawal and CFA (for each region/axis, individual data are presented as percentage of control animals). Within-subject data were analyzed using linear regression. CFA-treated, red (n = 9). (For interpretation of the references to colour in this figure legend, the reader is referred to the web version of this article.)

We have previously examined within-subject correlations in protein phosphorylation status across brain regions as a precursory marker of changes in inter-brain regional and possible circuit activity (Edwards et al., 2013). Utilizing this approach and analysis in the current study, we discovered significant positive correlations in GR phosphorylation levels between the insula and all other areas investigated (dmPFC, vmPFC, anterior cingulate cortex, and central amygdala) in animals exposed to both binge alcohol withdrawal and CFA (Table 1 and Fig. 6). These inter-brain regional correlations were not observed in control animals or in animals exposed to either condition alone.

**Table 1:** Inter-brain regional GR phosphorylation correlations in pain- & alcohol-experienced animals.

Treatment group	Insula-CeA	Insula-dmPFC	Insula-vmPFC	Insula-cingulate
Control/Saline	0.169	0.127	0.155	0.015
CFA/Saline	0.125	0.007	0.101	0.171
Control/Alcohol WD	0.204	0.029	0.024	0.339
CFA/Alcohol WD	0.825*	0.522*	0.519*	0.684*

## 4 Discussion

Based on the regular use of alcohol as an analgesic in individuals suffering from chronic pain (Brennan et al., 2005), as well as the emergence of hyperalgesia symptoms in many individuals suffering from alcohol withdrawal (Egli et al., 2012), this study investigated behavioral and neurobiological interactions between alcohol and chronic inflammatory pain. Chronic pain and alcohol use disorder (AUD) are characterized by similar behavioral attributes, including reward deficits, cognitive dysfunction, and enhanced negative affect (Edwards and Koob, 2010, Elman and Borsook, 2016). The profound negative emotional state generated by chronic pain is proposed to increase the risk of AUD development (LeBlanc et al., 2015). A chronic pain state may enhance the motivation to consume alcohol for several reasons. Alcohol intoxication may temporarily subdue negative affect due to the analgesic and anxiolytic action of alcohol, which could be further magnified by the reward of pain relief (Navratilova et al., 2012, Xie et al., 2014). In addition, both pain sensitivity and chronic pain-induced negative affect could be exacerbated in a state of alcohol withdrawal that in turn serves as a negative reinforcer to increase motivation for drinking (Apkarian et al., 2013, Edwards et al., 2020).

While initial reports in this area of research discovered the development of mechanical and thermal hyperalgesia in animal models of AUD (Dina et al., 2000, Edwards et al., 2012, Roltsch Hellard et al., 2017), more recent studies have attempted to understand how alcohol reward is altered in animal models of chronic pain. In comparison to the results of the present study in rats, a recent study of CFA-treated C57BL/6J male and female mice given continuous access to alcohol report that CFA-treated male (but not female) mice consume significantly more alcohol than control males when comparing the overall average of mean daily alcohol intake (Yu et al., 2019). However, another study examining CFA-treated mice to evaluate interactions between chronic pain and alcohol-drinking behavior reported no differences in alcohol consumption between groups or over time (Smith et al., 2015). In humans, empirical studies on how pain influences alcohol drinking have also yielded mixed results, although a confluence of studies appear to indicate that pain may be more likely to increase drinking and relapse in those who have began the transition to AUD (Brennan et al., 2005, Ekholm et al., 2009, Jakubczyk et al., 2016, Witkiewitz et al., 2015). Another transitory relationship exists between the analgesic efficacy of alcohol in humans relative to AUD status. While regular alcohol consumption is associated with reduced pain symptoms in most chronic pain sufferers (Ekholm et al., 2009; Macfarlane and Beasley, 2015), alcohol appears to increase pain and pain-related disability in problem drinkers and those with an AUD diagnosis (Brennan et al., 2005, Witkiewitz et al., 2015, Yeung et al., 2020). These findings warrant additional longitudinal studies examining how pain affects alcohol physiology in subjects transitioning from non-dependent to alcohol-dependent states to better understand how these relationships change over time.

In the current study, we first determined the behavioral consequences of chronic inflammatory pain in relation to alcohol drinking in non-dependent animals. Our initial hypothesis was that pain would increase alcohol self-administration, based on expectations that either inflammatory signaling (Blednov et al., 2012, Vetreno and Crews, 2014) or the negative affective state of pain itself would increase alcohol reward and drinking levels. To induce inflammatory pain, we administered a single injection of complete Freund’s adjuvant (CFA) that produces long-lasting hyperalgesia and increases negative affective-like behavior four weeks post-administration (Parent et al., 2012). Our findings indicate that the CFA model of inflammatory pain does not alter absolute levels of home cage alcohol consumption as measured by the intermittent-access two bottle-choice (2BC) alcohol drinking procedure in male Wistar rats (Fig. 1). During the initial phase of inflammation, and as inflammatory pain was sustained and became chronic, the amount of alcohol consumed by CFA-treated animals remained similar to that of control animals. Testing conducted at 1 week post-CFA revealed an inverse relationship between prior 24-hour alcohol drinking and thermal sensitivity (Fig. 2), appearing to reflect hyperalgesia produced in proportion to recent alcohol exposure. Although BALs were not determined at the time of cold plate testing due to potential stress-related confounds, several hours had elapsed since the termination of alcohol access, and BALs were likely near zero. This finding is consistent with the emergence of hyperalgesia symptoms during alcohol withdrawal that has been demonstrated across several animal models (e.g., Edwards et al., 2012). Over time, and possibly in association with the process of pain chronification, a surprising reversal in correlations between alcohol consumption and thermal sensitivity emerged, such that increasing amounts of alcohol consumption over the previous 24-hour drinking session were associated with increased paw withdrawal latencies (i.e., less thermal sensitivity) in CFA-treated animals when tested at 3 weeks post-CFA treatment. It could be speculated that the analgesic or rewarding efficacy of alcohol is increased over time in chronic pain states, although further experiments are required to directly examine this hypothesis.

A recent *meta*-analysis of alcohol-mediated analgesia in human subjects discovered a linear relationship between alcohol dose and analgesia, with blood alcohol levels that corresponded to binge-like alcohol exposure (0.08 mg/dL) producing a clinically relevant reduction in pain intensity (Thompson et al., 2017). Binge alcohol exposure is considered a primary risk factor for the eventual development of AUD based on its engagement of brain circuitry linked to negative reinforcement, including the central amygdala and prefrontal cortex (Gilpin and Roberto, 2012, Vargas et al., 2014, Pahng et al., 2017, Arienzo et al., 2019). The rebound negative affective consequences of acute binge alcohol exposure have been previously modeled in male Wistar rats. Acute (6hr) withdrawal from a single exposure to 2 g/kg alcohol produces increases in anxiety-like behavior (Zhang et al., 2007), brain reward thresholds (Schulteis and Liu, 2006), and conditioned place aversion (Morse et al., 2000). Such negative affective-like manifestations appear to represent behavioral adaptations that act in an opponent fashion to alcohol’s anxiolytic and other rewarding properties, and may foster early neurobiological mechanisms of negative reinforcement thought to underlie the transition to AUD (Edwards and Koob, 2010, Edwards, 2016). We adopted this model to examine how chronic pain alters neuroadaptations produced by binge alcohol exposure and withdrawal (reflecting possible neurobiological consequences for patients that may binge drink to cope with pain). Numerous preclinical models have demonstrated a functional role for the CeA in regulating persistent hyperalgesia associated with a range of neurological and psychiatric conditions ranging from arthritis (Ji et al., 2007) to opioid dependence (McNally and Akil, 2002). Also of particular interest is the role of the PFC in organizing behavioral goals (such as avoiding negative emotional conditions) in the context of nociception (Vogt, 2005a, Vogt, 2005b), and evidence for this intersection in frontocortical regions, including the cingulate cortex, has been extensively demonstrated in preclinical pain models (Johansen et al., 2001, Cao et al., 2009). Thus, we hypothesized that significant neurobiological interactions would exist between alcohol withdrawal and pain within the CeA and specific frontocortical areas as reflected by altered neuronal activity (measured via ERK phosphorylation) and stress-related signaling (indexed by GR phosphorylation).

Alcohol withdrawal produced widespread increases in ERK phosphorylation throughout multiple frontocortical areas, including the cingulate cortex, insula, and medial prefrontal cortex (Fig. 4). Interestingly, these neuroadaptations were blunted or absent in animals with a history of chronic inflammatory pain, indicating that chronic pain may produce a dampening of certain frontocortical responses to alcohol withdrawal. Chronic pain states are indeed associated with a characteristic loss of grey matter throughout the frontal cortex (Rodriguez-Raecke et al., 2009), although the functional consequences of pain-related PFC deficits for alcohol-related behaviors remains unclear and warrants further investigation. As frontocortical regions contain a mix of excitatory and inhibitory neurons, glial cells, and even top-down control of affective pain processes (Lee et al., 2015), additional studies will be necessary and vital to determine the cellular localization of this interaction, as well as relevant downstream circuit implications. For example, recent investigations have described an important role for the PFC in descending control of pain via connections to both the PAG and mesoaccumbens dopamine systems (Ong et al., 2019). Our findings of “tolerance” to alcohol withdrawal-induced ERK phosphorylation in the context of chronic pain may relate to a compromised engagement of these adaptive top-down processes that may ultimately worsen the combined negative affective experience of these two insults. Future circuit-based manipulations will be needed to examine this hypothesis. With specific regard to ERK signaling, chronic pain-induced negative affect is known to be mediated by increased activity of the GR-regulated MAPK-phosphatase (MKP-1) within at least the cingulate cortex (Barthas et al., 2017). As glucocorticoid signaling supports MKP-1 actions to inhibit ERK (Kassel et al., 2001), this mechanism would appear to remain intact as we did not observe tolerance to GR phosphorylation across frontocortical areas (Fig. 5).

Based on the importance of GR activity in both alcohol dependence and pain (Edwards et al., 2015), we also examined GR phosphorylation (pGR) as a marker of stress-related glucocorticoid signaling. We discovered that acute withdrawal from alcohol significantly increased GR phosphorylation throughout frontocortical regions, although as mentioned increased pGR was not modified by a state of chronic inflammatory pain. This finding may have important implications for individuals engaging in binge drinking, as even a single episode appears to engage stress-related signaling throughout vital frontocortical circuitry during the subsequent withdrawal (hangover) period. However, in contrast to our frontocortical findings and previous work demonstrating that chronically alcohol-dependent animals exhibit increases in CeA pGR (Vendruscolo et al., 2015), we did not see increases in CeA GR phosphorylation here after a single binge alcohol exposure and withdrawal. These data suggest that repeated bouts of binge alcohol intoxication and withdrawal are necessary to engender this neuroadaptation.

Interestingly, we did observe a striking congruence of within-subject, inter-brain regional correlations of GR phosphorylation only in animals experiencing both chronic pain and binge alcohol withdrawal (Fig. 6), with the insular cortex acting as a hub for these relationships with other nociceptive regions investigated. The insula contributes to the processing of sensory, cognitive, and affective dimensions of pain through its connectivity with the cingulate cortex, prefrontal cortex, and amygdala. The insula receives nociceptive information directly from the thalamus, and has reciprocal connections with the cingulate cortex as part of a network associated with pain sensation (Lu et al., 2016). The insula and anterior cingulate cortex are also key components of the salience network that facilitates the engagement of cognitive processes to a painful stimulus, and alterations in the salience network are observed in individuals with chronic pain and are associated specifically with greater pain catastrophizing (Kim et al., 2018). Insula connectivity with the amygdala is hypothesized to facilitate emotional arousal to painful stimuli (Moraga-Amaro and Stehberg, 2012, Lu et al., 2016). These circuit-based relationships have also been hypothesized to play a key role in the motivational processes relevant to AUD (Pahng et al., 2017).

Some additional factors warrant discussion. First, heavy alcohol drinkers often develop a characteristic form of small-fiber neuropathy (Diamond and Messing, 1994), and neuropathic (vs. inflammatory) pain mechanisms may more specifically alter alcohol drinking and reinforcement. In line with this hypothesis, sciatic nerve-ligated CD-1 mice consume more alcohol (20%) compared to sham-operated controls (Gonzalez-Sepulveda et al., 2016). More severe pain conditions, such as osteoarthritis, may also be necessary to alter drinking patterns. In this regard, Butler et al. (2017) discovered that surgically destabilizing the medial meniscus facilitated preference for alcohol (20%) in C57BL6/6J mice. Interestingly, the analgesic/anti-hyperalgesic efficacy of alcohol appears to be equal across both inflammatory and neuropathic pain conditions in mice (Neddenriep et al., 2019), and a greater understanding of the neurobiological mechanisms of alcohol analgesia, as well as analgesic tolerance, is needed (Cucinello-Ragland and Edwards, 2020). Chronic pain may also modify reward processes based on the analgesic used. Interestingly, opioid reinforcement appears to be altered in a very specific way such that lower doses are less reinforcing, while higher doses are more reinforcing (Hipolito et al., 2015). Finally, sex differences may play a considerable role in these relationships, including sex-dependent medication strategies for treating alcohol-induced hyperalgesia symptoms (Bergeson et al., 2016).

Finally, it is also important to note that self-administering animals in these tasks were not dependent upon alcohol. Functional neuroadaptations (including GR phosphorylation in the CeA) are known to manifest in the context of alcohol dependence that would not be present in non-dependent animals, even after an initial binge-like exposure (Fig. 4). Future studies will implement alcohol-dependent animals to determine if a state of preexisting dependence alters the impact of an additional painful experience. Interactions between pain and alcohol withdrawal are also likely to have implications beyond alcohol reinforcement, and our neurobiological findings could be important for frontocortical-related cognitive disruption associated with both chronic pain and binge drinking (Pahng et al., 2017, Carbia et al., 2018). Other brain areas impacted by nociception and pain-related behaviors may also be important in these relationships. Two examples not investigated here are the periaqueductal grey and the lateral habenula, which have been shown to mediate hyperalgesia symptoms during alcohol withdrawal (Avegno et al., 2018, Kang et al., 2019). We expect that continued examination of relevant circuits and neuroadaptations with central nociceptive areas will help shed light on new therapeutic avenues for treating both chronic pain and closely related motivational disorders such as AUD.

## Declaration of Competing Interest

The authors declare that they have no known competing financial interests or personal relationships that could have appeared to influence the work reported in this paper.
